# Diseases of the Nervous System

**Published:** 1896-03-21

**Authors:** 


### Progress in Medicine.

### DISEASES OF THE NERVOUS SYSTEM.

*(Continued from page 384.)*

**Anatomy and Physiology.**—Though limitations of space prevents our giving an outline of Ramon y Cajal's[13] article on "Evolution of the Nerve Cells," we would direct attention to it as being a valuable abstract of the subject utilising the most recent data.

"The Biological and Morphological Constitution of Ganglionic Cells, as Influenced by Section of the Spinal Nerve Roots or Spinal Nerves," a prize essay by Onuf,[14] affords some further information on this important question. He reminds us that one of the latest observers, Vanlair, has attained results which seem to contradict all observations made heretofore for Vanlair stated that unilateral section of the spinal cord, whether a regeneration of the latter took place or not, is in no case followed by any alterations in the structure of the spinal cord. After tabulating briefly the results of former observers, Onuf remarks that it is noteworthy that all cases in which the postero-lateral group of ganglion cells was affected were taken from the spinal cord of persons or animals on whom amputations or section of peripheric nerves had been performed, while in none of them had isolated section of the posterior or anterior roots been made. But, as he shows, there is reason to doubt that the postero-lateral group has sensory functions. It must also be kept in mind that in many cases of amputation or section of the sciatic nerve the principal alterations were found in the central or antero-internal or antero-lateral group. Accepting Friedlander and Krause's theory, these groups must therefore have sensory functions, a conclusion which contradicts too seriously the findings in progressive muscular atrophy, poliomyelitis anterior, &c., to be accepted. Now the author's present observations deal only with the changes noticed in the central (ascending) portion of severed motor fibres, not with secondary degeneration in the peripheric portion. To simplify observation, and in order to avoid complicating anatomical factors, Onuf selected the dorsal nerves for his experiments, operating on the eighth, ninth or tenth dorsal. He found that section of the anterior root of a dorsal nerve produced within four weeks a degeneration of the large multipolar cells of the anterior horns, cells to which we ascribe motor functions. This degeneration may be called a homogeneous degeneration for reasons given. The beginning stages of said degeneration are characterised by a swelling of all parts of the cells, &c. The author cites Howell and Huber, who found that after severance of nerve fibres from their connections with their nerve centres, "degeneration of the peripheral end was complete throughout the entire length. The least time in which irritability began to return to parts peripheral to the cut was twenty-one days, and at this time regeneration was found to have progressed some distance beyond the wound." He states that it is very probable that while the peripheral ends of the severed peripheral nerve-fibres undergo degeneration, retrogressive changes take place simultaneously in the nerve cells from which these fibres originate, and that when the fibres, or better said, the axis cylinders of the central stump, begin to grow into the peripheral stump, the ganglionic cells also begin to recover. The results of his own investigations lead to conclusions, which we give in part as follows: 1. The severance of the continuity of a spinal nerve, or of both of its roots, is always followed by retrogressive changes, both in cells which are intimately connected with its posterior roots, and in cells from which its motor fibres originate. 2. These changes may set in gradually, and consist in a gradual shrinkage of all parts of the cell, accompanied by a modification of its structure, or the changes may set in acutely. 3. The character of the retrogressive changes is determined (*a*) by the distance of the point of lesion from the cell. (*b*) By the character of the cell, or more probably by the manner of its connection with the severed fibres. In "motor" cells severance of the motor fibres near origin from the cell (section of the anterior root) is followed by the acute changes, viz., by homogeneous transformation of the cell. Lesion of the motor fibres at a considerable distance from the cells affects as a rule the gradual changes, that is a gradual shrinkage of the cell. 4. The gradual shrinkage gives, probably, much more chance for the recovery of the cell, if the continuity of the severed fibre can be restored, than the homogeneous degeneration.

**New Formation of Nerve Cells.**—A. N. Vitzov[15] found in the brain of a monkey a new formation occupying the back part of the skull, after incision of the occipital lobes two years previously. This mass was proved to contain nerve cells and neuroglia cells, exactly comparable in appearance to those present in normal brain tissue.

**Localisation of Functional Activity in Nerve Centres—Frontal Lobes.**—Demoor[16] has shown that the nerve cells of the brain of an animal poisoned with large doses of morphine or chloral hydrate exhibit remarkable changes in structure; thus the processes of cells became beaded or moniliform; and if the dose of morphine be large, they seem to be composed of beads, with excessively fine fibres uniting them to each other, presenting a resemblance to the amœboid prolongations of myxomycetes, or the protoplasmic processes of or within certain vegetable cells when excited, *e.g.*, by electricity. Fano,[17] at the third International Congress of Physiologists, after accurately determining the "latent period" or "response time" following faradic stimulation of a given motor area of the brain, then stimulated simultaneously the frontal lobes, and found that such stimulation was followed by increase of the latent period, while after removal of the frontal lobes (time being allowed for complete recovery) the latent period was diminished. As removal of the occipital lobes did not produce this effect, or only to a slight degree, Fano concludes that the frontal lobes are inhibitory in their action.

A very interesting case is recorded by Reagan[18] as follows: "Just before the close of the last war a company of Federal soldiers passed his home, the officer telling him that they had killed Lieutenant Smith some mile back. As soon as they had passed, Dr. Reagan searched for Smith and found him sitting in the woods fifty yards from the road. He was shot in the forehead; the bullet entered the left lobe of the brain; he was also shot in the side and right arm. His mind was clear, and remained so for two days and two nights, up to a few hours of his death. The brain worked out at the bullet hole so freely that after he had lost at least half an ounce of brain Reagan put plaster over the hole. Though he had been left for dead he had got up and walked some distance, and could talk and give all the particulars of the shooting, and talked up to a few hours of death. Reagan reports another case in his experience, in which a boy fifteen years old shot himself with a pistol, the bullet passing inside the skull near the nose, just missing the eye-ball. Though at first unconscious, with four respirations per minute, after recovering from the shock the mind was unaffected, but there was slight paralysis of the left arm and leg for a time.

**Splanchnic Nerves.**—Biedl[19] has shown experimentally that the splanchnic nerves are derived from ganglionic motor cells between the sixth cervical and fifth dorsal nerves, the cells in degenerating behaving like other motor cells in the anterior cornua after section of their peripheral nerves.

**Patellar Tendon Reflex.**—A case of diabetes with loss of knee-jerks, which reflex reappeared on the left side after the occurrence of Weber's syndrome, left hemiplegia with total incomplete paralysis of the right oculo-motor nerve, viz., ptosis with external strabismus and diplopia, dilatation of pupil, and paralysis of accommodation, is recorded by

Marinesco.[20] This observer remarks that the disappearance of the knee-jerks in diabetes has never been satisfactorily explained. If, in some cases, this abolition is due to lesions involving the centripetal fibres necessary for the transmission of the patellar reflex, its reappearance may be accounted for by an antagonistic action exerted on the anterior cornu by the pyramidal fibres on one hand, and the tendinous sensory fibre on the other. Under normal conditions the tendinous reflex tonus is maintained by continuous stimulation, determined by the tendinous sensory fibres and transmitted to the spinal cord through the reflex collaterals, while the pyramidal fibres exert a moderating influence on the excitability of the anterior cornu. When the tendinous centripetal fibres are diseased, the knee-jerk is diminished or abolished; but, if the moderating action of the brain ceases to exist, the tendinous centripetal fibres, which are still healthy, may in a measure re-establish the reflex tonus which previously appeared to be completely abolished. In connection with this case, we may recall a similar instance of recovery of a lost knee-jerk reported by Raichline,[21] at the Society of Biology, Paris, last June, that of a tabetic patient in whom secondary contracture with restoration of the tendon reflexes occurred after an organic hemiplegia in a person afflicted with tabes. This, he remarks, is an unusual event, as usually a hemiplegia remains placid, and the reflexes do not return if the person afflicted is already tabetic. He considered the loss of the knee-jerk in tabes as not due to a destruction of the reflex arc, but that the disease exerts an inhibitory influence on the reflex activity of the cord, and that, owing to the secondary degeneration of the crossed pyramidal tracts, this reflex activity is exaggerated, and the knee-jerk is thus restored in some cases. F. Edgeworth[22] recently showed a case of ataxic hemiplegia with exaggerated knee-jerks, an unusual phenomenon, which is readily explained in the light of the above-mentioned cases, while the fact that in ataxic hemiplegia the knee-jerk is usually absent is probably owing to the interference with the centripetal fibres of the reflex arc being too pronounced for the exaggeration due to the lateral tract degeneration to have any evident effect as regards the knee-jerk.

[13] Journ. of Nervous and Mental Diseases, Dec., 1895. [14] Ibid., Oct., 1895. [15] Cited from Med. Chron., Nov., 1895. [16] Ibid. [17] Ibid. [18] Charlotte Med. Journ., Nov., 1895. [19] Med. Week, Dec. 13, 1895. [20] Ibid., Nov. 1. [21] Intern. Med. Mag., No. 10, 1895. [22] Bristol Med. Chi. Soc., Mar. 11, 1896.

